# Bacteriocin-Producing *Escherichia coli* Isolated from the Gastrointestinal Tract of Farm Animals: Prevalence, Molecular Characterization and Potential for Application

**DOI:** 10.3390/microorganisms10081558

**Published:** 2022-08-02

**Authors:** Marina V. Kuznetsova, Veronika S. Mihailovskaya, Natalia B. Remezovskaya, Marjanca Starčič Erjavec

**Affiliations:** 1 Institute of Ecology and Genetics of Microorganisms, Ural Branch of the Russian Academy of Sciences, Goleva Street 13, 614081 Perm, Russia; mar19719@yandex.ru (M.V.K.); veranikamihailovskaja@yandex.ru (V.S.M.); natali.rem@yandex.ru (N.B.R.); 2 Department of Microbiology, Biotechnical Faculty, University of Ljubljana, Jamnikarjeva 101, 1000 Ljubljana, Slovenia

**Keywords:** *Escherichia coli*, antibiotics, probiotics, bacteriocins, colicins, microcins, farm animals

## Abstract

Due to the spread of antibiotic-resistant bacteria, new alternatives to antibiotics and ways to prevent infections are being sought. Bacteriocin-producing bacteria are therefore attracting attention due to their probiotic potential as a safe alternative to antimicrobial drugs. The aim of this work was to determine the prevalence of bacteriocin-encoded genes among *Escherichia coli* strains from healthy farm animals and to characterize the presence of virulence-associated genes, the possibility of prophage induction, and hemolytic and bacterial antagonistic activity of the bacteriocin-producing *E. coli* in order to reveal their potential for application. It was found that 17 of 72 *E. coli* strains (23.6%) produced bacteriocins. Among them, 18 out of 30 bacteriocin genes were detected: the most prevalent genes were those for microcin M (58.8%), colicin E1 (52.9%), and colicin M (35.3%). Colicin Ia (29.4%), colicin E9, colicin Ib, colicin B (23.5%), and colicin E9 (17.7%) genes were also frequent, while the prevalence of genes encoding microcins V, B17, and H47 and colicins E3, K, N, U, Y, 5, and 10 did not exceed 11.8%. At least two different bacteriocin genes were detected in all 17 bacteriocinogenic strains; the highest number of different bacteriocin genes detected in one strain was seven genes. *E. coli* strains with combinations of colicin E1 and E or microcin M and colicin E1 genes were more prevalent than others (17.7%). Among the 17 bacteriocin-producing *E. coli* strains, 5.9% were hemolytic, 47.1% contained prophages, and 58.8% carried genes encoding toxins. Cell-free supernatants of bacteriocin-producing strains were shown to inhibit the growth of pathogenic *E. coli* strains belonging to the APEC, STEC, and ETEC pathotypes. Thus, among the studied bacteriocin-producing *E. coli* isolated from the gastrointestinal tract of farm animals, three strains with high antagonistic bacterial activity and the absence of pathogenicity genes, prophages, and hemolytic activity were identified and therefore have potential for application.

## 1. Introduction

The intestinal microbiota of animals is a dynamic community of microorganisms, among which bacteria of the species *Escherichia coli* are of particular importance. Pathogenic strains of *E. coli* cause a wide range of diseases in livestock: intestinal pathogenic *E. coli* (IPEC) cause outbreaks of intestinal infections and uropathogenic *E. coli* (UPEC), sepsis-causing *E. coli* (SePEC), and neonatal meningitis-associated *E. coli* (NMEC) lead to infectious processes in extra-intestinal areas [1,2]. Of special significance are avian pathogenic *E. coli* (APEC) which cause colibacillosis [3,4]. Commensal *E. coli* produce vitamins, are involved in modulating the host immune system, strengthen the intestinal barrier, and provide colonization resistance of the intestinal mucosa against pathogenic and opportunistic bacteria competing for a place in this ecological niche [5,6].

One of the main mechanisms of the antagonistic activity of commensal *E. coli* is the secretion of antimicrobial peptides/bacteriocins [7]. *E. coli* produce two types of bacteriocins: colicins and microcins [7,8]. Bacteriocins have highly specific activity against phylogenetically related species and unique mechanisms of action [9]. Some colicins form pores (colicins A, E1, K, N, U, S4, B, Ia, Ib), some have nuclease activity, for example, DNase (E2, E7, E8 and E9), 16S RNase (E3, E4, E6), and tRNase (E5, D), and colicin M inhibits the biosynthesis of peptidoglycan [7,10]. Microcins are able to form pores in the bacterial membrane (microcins V and L), inhibit DNA gyrase (B17), RNA polymerase (J25), and disrupt ATP synthase (H47) [10,11].

Since the widespread use of antimicrobials, including in agriculture, has led to the spread of antibiotic resistance, studies are being conducted on the antimicrobial activity of bacteriocins and bacteriocin-producing strains as an alternative to antibiotic therapy [7,12,13,14]. On the one hand, bacteriocin-producing strains can be used as producers of new antimicrobial substances/bacteriocins; on the other hand, bacteriocin-producing strains themselves can be used as probiotics. The main staples in the search for new useful bacteriocin-producing *E. coli* strains are the isolation of bacteriocin-producing bacteria from natural sources, the comparative assessment of their activity, and the selection of the most promising strains [15,16]. *E. coli* from the normal gut microbiota of healthy animals are one of the safe sources of bacteriocin-producing strains.

The aim of the study was to determine the prevalence of bacteriocin-encoded genes among *E. coli* strains from the feces of healthy farm animals and to characterize the presence of virulence-associated genes, the possibility of prophage induction, and the hemolytic and bacterial antagonistic activity of the bacteriocin-producing *E. coli* in order to reveal their potential for application.

## 2. Materials and Methods

### 2.1. Bacterial Strains

For this study, 97 *E. coli* isolates obtained from fecal samples of healthy animals (chicken, turkeys, pigs, rabbits, cows) at agricultural enterprises and private farms in Perm Krai (Russia) between 2019 and 2021 were used (Table 1). The diluted animal feces was sown directly onto the surface of the MacConkey agar (Sigma-Aldrich, St. Louis, MO, USA), plates were incubated overnight at 37 °C, and the next day typical *E. coli* colonies (pink colored, transparent smooth and raised colonies or dark red colonies with pronounced metallic luster) were taken from the MacConkey agar and subcultured onto MacConkey agar again. Gram staining (Gram-negative rods) and biochemical tests: Kligler Iron Agar (decomposes glucose and lactose to acid and gas, without hydrogen sulfide–yellow color with interruptions); Simmons Citrate Agar (does not use citrate-not change the color); and catalase (positive) and oxidase tests (negative) were used for preliminary identification of bacteria. Finally, for confirmation of *E. coli* by the ENTEROtest 16 diagnostic system (Erba Lachema s.r.o., Czech Republic) with the Russian version of the Micro-La-test “Microb-2” (Microbiological system monitoring “Microbe-2”, SMMM-2) computer program, one randomly chosen isolate from each animal, thus 97 isolates in total, were taken. Isolates with an “acceptable” rating and a match rate of more than 94% were confirmed as *E. coli*. All 97 by ENTEROtest 16 analyzed isolates were confirmed to be *E. coli*, so all were taken for the final differentiation between clonal and non-clonal *E. coli* with ERIC-PCR [17]. Finally, 72 non-clonal *E. coli* strains (6 strains from chicken, 3 from turkey, 7 from quail, 3 from pigs, 4 from rabbits, 49 from cows) were collected and further analyzed in this study.

To assess the antagonistic effect, the following strains were used: APEC strains (BR4, BR35, and BR37) were previously isolated from poultry with colibacillosis at Perm Krai breeding farms [18] and deposited in the Ex culture collection of the University of Ljubljana (Univerza v Ljubljana, Slovenia) under the numbers L-5838, L-5865, and L-5868, respectively. IPEC strains (CA29, CA43, CA46) were obtained from cattle at agricultural enterprises in the Perm Krai. The belonging of test-strains to IPEC pathotypes was determined by detecting STEC and ETEC marker genes: *stx1* (strain CA29), *est1* (strain CA43), *stx2+est1* (strain CA46). The resistance of test strains to antibiotics was determined by the disc-diffusion method [19].

### 2.2. Prevalence of Bacteriocin-Producing Strains

The detection of bacteriocin-producing strains was performed with the agar overlay method according to M. Budič et al., 2011 [20]. *E. coli* DH5α (SCPM-Obolensk, State Collection of Pathogenic Microorganisms and Cell Cultures of the State Research Center for Applied Microbiology and Biotechnology) was used as the sensitive indicator strain and *E. coli* ŽP, carrying the colicin E7 gene, constructed at the Biotechnical Faculty of the University of Ljubljana on the basis of the probiotic strain *E. coli* Nissle 1917 [21], was used as the bacteriocin-producing positive control strain. Strains that induced a lysis zone around the spotted tested isolated *E. coli* colony in the overlaid indicator strain were recognized as bacteriocin-producing. The diameters of the lysis zones of the indicator strain were measured (mm).

### 2.3. Molecular Characterization

#### 2.3.1. DNA Extraction

To obtain matrix DNA for PCR amplification, a loop of bacterial biomass was resuspended into 100 µL of ultrapure water, heated for 15 min at 97 °C in a solid-state thermostat with a timer TT-2 “Termite” (Russia), and centrifuged for 5 min at 13,000 rpm. The supernatants were transferred to fresh Eppendorf tubes and stored at −20 °C until further usage.

#### 2.3.2. Detection of Genes Encoding Bacteriocins

Bacteriocin-producing strains were further characterized for bacteriocin genes using PCR-based screening, and 30 genes encoding bacteriocins (microcins B17, H47, J25, V, M, C7, L; colicins-A, B, D, E1, E2, E3, E4, E5, E6, E7, E8, E9, Ia, Ib, Js, K, M, N, S4, U, Y, 5, 10) were detected by PCR. PCR reactions were carried out in 25 µL of the reaction mixture using primers and amplification conditions according to the authors’ recommendations [8,22]. Amplifications were performed in PCR mixtures with Taq-polymerase (LLC “Sintol”, Moscow, Russia) in a thermal cycler DNA Engine Dyad Thermal Cycler (“Bio-Rad”, Foster City, CA, USA). Gel documentation system Gel-DocXR (“Bio-Rad”, USA) was used for band visualization and data documentation.

#### 2.3.3. Detection of Virulence-Associated Genes

All detected bacteriocin-producing *E. coli* strains were analyzed for the presence of 22 virulence-associated genes encoding either toxins (*hlyA,* *hlyF*, *east1,* *ehxA,* *estI,estII,* *eltA,* *stx1,* *stx2,* *cnf1*) or adhesins (*fimH,* *papC,* *afa/draBC*) with specific primers and amplification programs, according to recommendations of the authors [23,24]. PCR mixtures used and equipment are already stated above.

### 2.4. Potential for Appliaction

#### 2.4.1. Preparation of Cell-Free Supernatants (CFS) of Bacteriocin-Producing *E. coli*

Bacteriocin-producing strains were cultured overnight in liquid Luria–Bertani medium (LB medium, “Difco”, Le Pont de Claix Saint-Ferréol, France) at 37 °C without aeration. The grown bacterial cultures were transferred into Eppendorf tubes and centrifuged for 10 min at 13,000 rpm. The supernatants were sterilized using Millex^®^-GS membrane filters (“Merck Milli-pore Ltd. “, Tullagreen, Carrigtwohill, County Cork, Ireland) with a pore diameter of 0.22 microns. CFS were stored at −20 °C.

#### 2.4.2. Evaluation of Antagonistic Activity of CFS of Bacteriocin-Producing *E. coli*

The antagonistic effect of bacteriocin-producing strains was assessed by evaluating the bacterial growth of pathogenic *E. coli* (APEC and IPEC) in the presence of metabolites of the studied *E. coli* strains in the culture medium. Overnight LB broth (“Difco”, Le Pont de Claix Saint-Ferréol, France) cultures of APEC and IPEC were adjusted to 10^6^ cell/mL with fresh medium, and 100 µL were added to the wells of 96-well microtiter plates containing either 100 μL CFS from 24 h cultures of bacteriocin-producing *E. coli* (antagonist strains) or 100 μL of fresh LB (control wells). The microtiter plates were incubated without shaking for 24 h at 35 °C. Subsequetly, the OD_600_ of cultures was measured using the plate reader INFINITE M1000 (Tecan Austria GmbH, Grödig, Austria, and the percentage of growth inhibition after 22 h of co-cultivation was calculated, taking as 100% the optical density of the culture grown in the control wells.

#### 2.4.3. Bacteriophage Induction

Bacterial overnight cultures were diluted in saline phosphate buffer (PBS) in order to obtain a concentration from 1 × 10^5^ to 1 × 10^6^ bacteria per ml, and 20 mL of such diluted overnight cultures were transferred into standard Petri dishes for exposure to the continuous UV-light treatment (λ = 260) for 70 s. After the UV exposure, the cultures were incubated for 1 h at 37 °C and then mixed with a culture of the sensitive strain *E. coli* DH5α and added to melted 0.6% agar (46 °C), mixed, and poured onto LB agar plates. After a 24 h incubation at 37 °C the presence of lysis zones of the sensitive strain was screened for.

#### 2.4.4. Hemolytic Activities

The hemolytic activities of farm strains were determined by streak-plating on ram blood agar 5% (*v*/*v*; “Biomed“ a branch of FSUE “Microgen“, Russia). After a 24 h incubation at 37 °C the hemolytic zones were observed and the diameter (mm) of the lysis zone was measured.

### 2.5. Statistical Analysis

Data are presented as the arithmetic mean and its standard deviation (M ± SE). The statistical significance was tested with Fisher’s exact test or Student’s test (*t*-test), and *p*-values < 0.05 were considered statistically significant. Data processing was carried out using computer programs Microsoft Office XP Excel and STATISTICA 10.0.

## 3. Results

### 3.1. Prevalence of Bacteriocin-Producing E. coli

Among the 72 *E. coli* strains studied, 17 (23.6%) were capable of producing lysis zones in the overlaid indicator strain DH5α, and hence these strains were recognized as bacteriocin-producing strains. The majority of these strains were isolated from fecal samples of cows (26.5%), while bacteriocin producers were not detected among fecal isolates from turkeys, rabbits, and pigs. It should be noted that among the tested strains, six bacteriocin-producing strains had lysis zone diameters larger than the bacteriocin-producing positive control strain ŽP (Table 2).

With PCR, 30 different bacteriocin genes were screened for. Among the recognized 17 bacteriocin-producing *E. coli* strains, 18 of the screened 30 bacteriocin genes were found (Table 3). In all, in 17 strains at least one bacteriocin gene was found, and 47.1% of strains possessed two genes, 17.7% possessed three genes, and 29.4% of strains possessed four or more genes. One strain possessed six and one strain possessed seven different bacteriocin genes. 41.2% of the strains contained only colicin genes, and 58.8% contained both colicins and microcins.

Among the studied bacteriocin-producing strains, the most prevalent were microcin M (58.8%) and colicin E1 (52.9%) genes, followed by colicin E9, colicin Ib, colicin B genes (all three found in 23.5% of strains), colicin Ia genes (found in 29.4%), and colicin M genes (in 35.3% of bacteriocin-producing strains). The genes of microcins B17 and V and colicins K, E8, U, and Y were found each only in one strain. The genes of microcins J25, C7, and L and colicins E2, E4, E5, E6, E7, A, D, S4, and Js were not detected. Of the 17 strains, 12 (70.6%) had unique profiles of bacteriocin genes (not repeated in any other analyzed strain). *E. coli* strains with combinations of colicin E1 and E9 genes or microcin M and colicin E1 genes were each found in 17.7% of strains.

**Figure 1:**
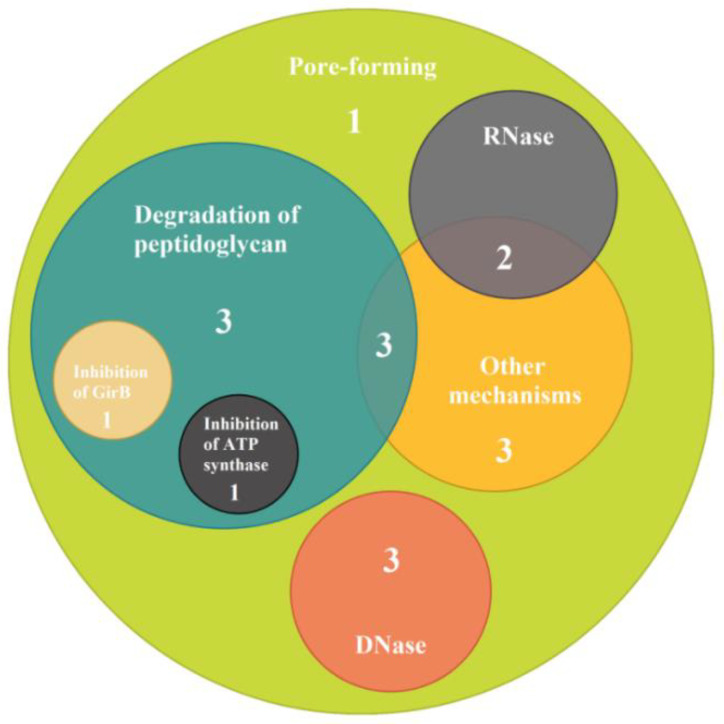
Combinations of bacteriocin genes with different modes of actions (the number reflects the number of strains with corresponding combinations of modes of actions).

All analyzed bacteriocin-producing strains encoded at least one pore-forming bacteriocin (Figure 1). The gene of colicin M with the mode of action of peptidoglycan degradation was found in 35.3% of strains. Strains encoding colicins and microcins with two different modes of action were found in 58.8% of strains, followed by those encoding bacteriocins with three different modes of action and four different modes of action, found in 23.5%, and 11.8% of strains, respectively. The probability of detecting strains containing genes of several bacteriocins with the same mode of action was only 5.9%; 35.3% of strains encoded simultaneously bacteriocins with a pore-forming mechanism and with the mode of action of degrading the peptidoglycan, and 29.4% of strains encoded simultaneously bacteriocins with a pore-forming mechanism and bacteriocins degrading nucleic acids. Strains that had *E. coli* DH5a lysis zone diameters of 10 mm or more often contained combinations of four or more bacteriocin genes (Fisher’s exact test: *p* = 0.02).

### 3.2. The Effect of CFS of Bacteriocin-Producing E. coli on the Growth of Pathogenic E. coli

All CFS obtained from the 17 bacteriocin-producing *E. coli* strains inhibited the growth of pathogenic *E. coli* after 22 h of cultivation, albeit with different levels of efficiency (Table 4). It should be noted that the antagonistic activity of most of the studied strains was higher than that of the control ŽP strain against APEC strains and CA29.

The maximum value of inhibition index was 58.6% (CFS obtained from strain C23 on BR4 and CFS obtained from C41 on multidrug resistant BR35). All CFS effectively suppressed the growth of the multidrug resistant BR37; however, the maximum inhibition index reached 47.6% (suspension of strain Q8). In the presence of CFS, growth reduction of IPEC strains isolated from cattle was also observed, although the obtained inhibition indexes were smaller than for APEC: for most strains the difference was statistically significant (with the exception of strains C41, C48, and C61 (*t*-test)). For the hetero-pathogenic strain CA29, the maximum inhibition index was 36.6% (CFS of strain C61). The addition of CFS of strain CA49 did not inhibit the growth of pathogenic strains C43 and CA46. The CFS of strain CA61 had the maximum inhibition index (42.0% for the C43 and 42.1% for the C46). It should be noted that the antagonistic effect was observed against strains of both pathotypes, APEC and IPEC, and also against multidrug resistant strains (Table 5).

### 3.3. Biological and Molecular-Genetic Properties of Bacteriocin-Producing E. coli Strains

The biological characteristics of bacteriocin-producing *E. coli* strains are presented in Table 6. Only the strain C45 was detected to be hemolytic, while 47.1% of strains possessed prophages that could be activated by UV. Virulence-associated genes encoding toxins were carried by 58.8% of strains. Three strains (Q5, C23, and C41) did not contain any virulence-associated genes, nor bacteriophages.

## 4. Discussion

In this work, screening of bacteriocin-producing *E. coli* strains isolated from healthy farm animals was performed as well as further characterization of the bacteriocin-producing *E. coli* strains found: the detection of bacteriocin genes and virulence-associated genes. In addition, CFS of the bacteriocin-producing strains were tested for antagonistic activity against APEC and IPEC, including multidrug-resistant strains.

The percentage of bacteriocin-producing *E. coli* strains isolated from different biotopes and ecological niches varied in different studies. In the study by Cameron et al., 2019 [15], 15.6% of isolates isolated from wastewater and feces of cattle inhibited the growth of the test culture. Mazurek-Popczyk et al., 2020 [7], showed that among isolates obtained from the feces of healthy humans, 37.1% were bacteriocinogenic. Micenková et al., 2016 [25], reported much higher numbers: 54.2% of all tested fecal *E. coli* isolates were found to be bacteriocin producers. An even higher prevalence of bacteriocin producers was shown by Budič et al., 2011 [20], in the population of *E. coli* isolated from patients with bacteremia: 61% of the strains carried at least one bacteriocin. In our study, the prevalence of bacteriocin-producing *E. coli* strains among representatives of the microbiota of healthy animals was 23.6%, while among cattle the frequency of occurrence of such strains was 26.5%. The relatively low prevalence of bacteriocinogenity in the population of *E. coli* obtained from healthy farm animals might be explained by the conditions in agricultural enterprises—the animals are artificially isolated from the environment (mostly kept indoors, use of standard feeding). Indirectly, this assumption could be confirmed by the fact that mainly bacteriocin-producing strains were isolated from cows, grazing outdoors in the summer–autumn period, while bacteriocin-producing strains were not isolated from turkeys, rabbits, and pigs living in boxes/cages all year round. Another fact that limits the biodiversity of strains, including bacteriocinogenity, may be the use of multiple antibiotic therapy regimens at the enterprises.

It is known that encoding multiple bacteriocins gives the cell a selective advantage since such strains have a broader inhibitory effect spectrum on competing organisms [11], including bacteria with multiple resistance to bacteriocins [26,27]. In a study by Gordon O’Brien, 2006 [8], it was shown that among the bacteriocin-producing strains, 42% produced one type of bacteriocin, 41% produced two, 16% produced three, and one strain produced four different types of bacteriocins. In our study, more than one bacteriocin gene was detected in all 17 strains, while about 30% of the strains had four or more bacteriocin genes. 

Among the *E. coli* strains isolated from different sources, most often genes of the following colicins E1, Ib, K, and M and microcins H47, M, and V were found [7,15,20]. A number of researchers have reported correlations of encoding microcins H47 and M as well as colicins B and M [8,28]. In our study, the majority of strains (94.1%) encoded several bacteriocins with different modes of action. The most prevalent were strains encoding pore-forming bacteriocins (colicin E1, Ia, B) and those encoding colicin M with the mode of action of peptidoglycan degradation as well as microcin M (Table 2, Figure 1). It should be noted that out of 30 bacteriocin genes, 12 were not detected, including the E7 colicin gene, which, according to Mazurek-Popczyk et al., 2020 [7], is a rare colicin among the commensal microbiota.

According to some studies, *E. coli* bacteriocins are effective against IPEC strains, including the O157:H7 serotype [29]. The development of probiotics based on bacteriocin-producing strains that eliminate STEC in farm animals can prevent zoonotic transmission of resistant bacteria to humans. Taking into account the problem of bacterial antibiotic resistance, bacteriocins are considered promising candidates for use in agriculture, especially in relation to multidrug-resistant bacteria [14]. Mazurek-Popczyk et al., 2020, showed that bacteriocin-producing *E. coli* strains showed the same antagonistic activity against antibiotic-resistant and antibiotic-sensitive zoonotic *E. coli* strains [7]. Our study also showed that CFS of bacteriocin-producing strains inhibited the growth of both sensitive and antibacterial-drug-resistant, even multidrug-resistant, pathogenic *E. coli* belonging to different pathotypes.

Interestingly, we did not find a significant difference in antagonistic activity against APEC strains that differ in sensitivity to various antibiotics and bacteriocins (Table 5). The CFS of *E. coli* strains C32, C40, and C41, encoding the colicin E9 with the DNase activity, inhibited growth of all APEC strains by 50% or more. The growth of strains BR4, BR35, and BR37, which differed in sensitivity to bacteriocins, was significantly suppressed in the presence of CFS of bacteriocin-producing *E. coli* from farm animals. This could be due to the action of other bacteriocins with different modes of action preventing the appearance of insensitive strains. It is known that the use of only one bacteriocin can quickly give rise to insensitive strains [20]. 

The main primary requirements for the selection and verification of probiotic strains of microorganisms are the absence of virulence-associated genes (the strain should not produce enzymes related to virulence) and mechanisms associated with horizontal transmission of genetic information [30]. Preliminary characterization of the strains in terms of hemolytic activity, lysogenicity, and the presence of virulence-associated genes allowed us to select three potentially probiotic strains with high antagonistic activity against pathogenic and conditionally pathogenic *E. coli* from the 17 bacteriocin-producing strains, i.e., studied strains Q5, C23, and C41, whose probiotic potential can be further explored. For the development of a probiotic product, this primary study should be followed up in the future with the evaluation of the strains’ ability to actively colonize and persist long-term in the biotope as well as their biological safety. It is also necessary to select the optimal growth medium for the biotechnological process of producing the potential probiotic drug and to evaluate the survival of the microorganisms in the form in which the drug will be stored.

## 5. Conclusions

Increasing resistance of microorganisms to antibiotics in different areas such as medicine and veterinary medicine has encouraged researchers to seek alternative antimicrobial therapies. The potential of commensal strains from healthy animals to defend against zoonotic pathogenic strains was estimated in this study. The study revealed that bacteriocinogenic commensal *E. coli* strains show antagonistic activity against IPEC and APEC strains and inhibited the growth of both sensitive and antimicrobial-drug-resistant pathogenic *E. coli* of animal origin belonging to different pathotypes.

## Figures and Tables

**Table 1:** Isolates and strains of *E. coli* isolated from farm animals.

Year	Number of Isolates (Strains) Isolated from the Source					
	Chicken	Turkey	Quail	Pig	Rabbit	Cow
2019	0 (0)	0 (0)	0 (0)	0 (0)	0 (0)	5 (1)
2020	7 (6)	4 (3)	12 (7)	5 (3)	7 (4)	18 (12)
2021	0 (0)	0 (0)	0 (0)	0 (0)	0 (0)	39 (36)

**Table 2:** Screening for the bacteriocin-producing *E. coli* by the agar overlay method.

Strain	Source	Diameter of *E. coli* DH5 α lysis Zone, mm
ŽP	Not applicable	6.0 ± 0.8
Ch1	chicken	8.5 ± 1.6
Q5	quail	13.0 ± 1.6 *
Q8	quail	8.0 ± 2.2
Q12	quail	6.5 ± 1.3
C18	cow	9.3 ± 1.0
C19	cow	12.3 ± 2.5 *
C23	cow	6.0 ± 1.4
C25	cow	18.8 ± 1.5 *
C32	cow	7.5 ± 1.0
C40	cow	7.0 ± 0.8
C41	cow	7.8 ± 0.5
C45	cow	8.0 ± 0.0
C48	cow	8.3 ± 1.0
C49	cow	7.3 ± 0.5
C51	cow	10.0 ± 1.4 *
C56	cow	13.8 ± 2.5 *
C61	cow	10.0 ± 0.0 *

Note: *—statistically significantly larger lysis zone in comparison with the lysis zone of the ŽP strain (*t*-test, *p* ˂ 0.05).

**Table 3:** Distribution and prevalence of bacteriocin genes among *E. coli* strains obtained from healthy animals.

Mode of Action of Bacteriocins	Bacteriocin Type	Strain																	Prevalence, %
		Ch1	Q5	Q8	Q12	C18	C19	C23	C25	C32	C40	C41	C45	C48	C49	C51	C56	C61	
Pore-forming	mccV	−	−	−	−	−	−	−	−	−	−	−	−	−	−	+	−	−	5.9
	mccL	−	−	−	−	−	−	−	−	−	−	−	−	−	−	−	−	−	0.0
	Ia	+	+	−	−	+	−	−	−	−	−	−	−	−	−	+	+	−	29.4
	Ib	−	+	+	+	−	−	−	−	−	−	−	−	−	−	−	−	+	23.5
	E1	+	−	−	−	−	−	−	+	+	+	+	+	+	+	+	−	−	52.9
	B	+	−	−	−	−	−	+	+	−	−	−	−	−	−	−	−	+	23.5
	K	−	−	−	−	+	−	−	−	−	−	−	−	−	−	−	−	−	5.9
	A	−	−	−	−	−	−	−	−	−	−	−	−	−	−	−	−	−	0.0
	N	+	−	−	−	−	−	−	+	−	−	−	−	−	−	−	−	−	11.8
	U	−	+	−	−	−	−	−	−	−	−	−	−	−	−	−	−	−	5.9
	Y	−	+	−	−	−	−	−	−	−	−	−	−	−	−	−	−	−	5.9
	S4	−	−	−	−	−	−	−	−	−	−	−	−	−	−	−	−	−	0.0
	5	−	−	−	−	+	+	−	−	−	−	−	−	−	−	−	−	−	11.8
	10	−	−	−	−	+	+	−	−	−	−	−	−	−	−	−	−	−	11.8
DNase	E2	−	−	−	−	−	−	−	−	−	−	−	−	−	−	−	−	−	0.0
	E7	−	−	−	−	−	−	−	−	−	−	−	−	−	−	−	−	−	0.0
	E8	−	−	−	−	−	−	−	−	−	−	−	−	−	−	−	−	−	0.0
	E9	−	−	−	−	−	−	−	−	+	+	+	−	−	−	−	−	−	17.7
RNase	E3	−	+	−	−	−	−	−	−	−	−	−	−	−	−	+	−	−	11.8
	E4	−	−	−	−	−	−	−	−	−	−	−	−	−	−	−	−	−	0.0
	E5	−	−	−	−	−	−	−	−	−	−	−	−	−	−	−	−	−	0.0
	E6	−	−	−	−	−	−	−	−	−	−	−	−	−	−	−	−	−	0.0
	D	−	−	−	−	−	−	−	−	−	−	−	−	−	−	−	−	−	0.0
Inhibition of DNA gyrase	mccB17	+	−	−	−	−	−	−	−	−	−	−	−	−	−	−	−	−	5.9
Inhibition of RNA polymerase	mccJ25	−	−	−	−	−	−	−	−	−	−	−	−	−	−	−	−	−	0.0
Degradation of peptidoglycan	M	+	−	+	+	−	−	+	+	−	−	−	−	−	−	−	−	+	35.3
Inhibition of Asp-tRNA synthesis	mccC7	−	−	−	−	−	−	−	−	−	−	−	−	−	−	−	−	−	0.0
Inhibition of ATP synthase	mccH47	−	−	−	−	−	−	−	+	−	−	−	−	−	−	−	+	−	11.8
Other mechanisms	mccM	+	+	−	−	−	+	+	+	−	−	−	+	+	+	+	+	−	58.8
	Js	−	−	−	−	−	−	−	−	-−	-−	−	−	−	−	−	−	−	0.0

Note: “+”—the gene was detected; “−“—the gene was not detected.

**Table 4:** Inhibition of the growth of pathogenic *E. coli* in the presence of CFS of bacteriocin-producing *E. coli*.

Strain	Inhibition Index, %					
	APEC			IPEC		
	BR4	BR35	BR37	CA29	CA43	CA46
ŽP	9.2 ± 6.2	5.6 ± 9.6	6.1 ± 7.9	1.7 ± 1.7	8.1 ± 7.6	0.8 ± 1.4
Ch1	31.5 ± 15.8	46.1 ± 9.0 *	37.2 ± 6.0 *	17.2 ± 0.4 *	19.8 ± 6.6 *	20.8 ± 6.7 *
Q5	38.3 ± 11.0 *	49.9 ± 7.3 *	41.3 ± 8.3 *	22.8 ± 4.5 *	29.9 ± 3.2	31.0 ± 3.7 *
Q8	47.1 ± 8.8 *	55.0 ± 7.8 *	47.6 ± 3.6 *	20.0 ± 5.9 *	23.4 ± 4.7	24.1 ± 4.2 *
Q12	28.4 ± 6.3 *	38.9 ± 3.8 *	42.7 ± 5.1 *	24.8 ± 3.2 *	17.0 ± 6.0	17.0 ± 5.3 *
C18	41.3 ± 11.8 *	44.8 ± 3.1 *	35.5 ± 9.3	22.7 ± 1.5 *	11.4 ± 2.1	12.7 ± 1.4 *
C19	30.6 ± 7.5 *	44.2 ± 3.9 *	31.5 ± 5.7 *	24.1 ± 3.1 *	14.8 ± 3.0	15.7 ± 1.6 *
C23	58.6 ± 11.5 *	51.7 ± 11.2 *	43.8 ± 3.9 *	10.4 ± 1.7 *	2.7 ± 2.7	3.6 ± 2.3
C25	34.4 ± 7.5	35.6 ± 4.1	36.9 ± 5.6 *	7.8 ± 2.9 *	6.3 ± 2.6	7.1 ± 1.2 *
C32	42.8 ± 10.4 *	51.6 ± 1.9 *	42.1 ± 4.6 *	20.2 ± 2.6 *	33.9 ± 7.8	32.5 ± 8.6 *
C40	45.4 ± 5.7 *	51.8 ± 3.5 *	40.0 ± 4.2 *	19.8 ± 0.7 *	11.8 ± 4.6	12.3 ± 4.7
C41	46.9 ± 17.6 *	58.6 ± 6.9 *	43.2 ± 6.9 *	24.3 ± 5.9 *	38.5 ± 5.7	36.8 ± 5.9 *
C45	27.6 ± 3.7	34.6 ± 2.9 *	38.2 ± 2.8 *	15.4 ± 1.7 *	3.6 ± 3.1	4.5 ± 3.4
C48	14.8 ± 16.0	31.0 ± 6.0	42.3 ± 5.3 *	17.4 ± 2.6 *	1.4 ± 2.4	3.0 ± 2.6
C49	40.9 ± 12.2 *	47.4 ± 3.6 *	34.3 ± 6.0 *	16.6 ± 2.4 *	0	0
C51	33.7 ± 10.6	41.1 ± 3.8 *	34.8 ± 5.1	18.5 ± 3.1 *	7.4 ± 6.6	7.8 ± 6.1
C56	42.4 ± 4.9 *	48.8 ± 4.1 *	38.1 ± 2.7 *	25.7 ± 4.0 *	21.2 ± 3.5	21.4 ± 3.2 *
C61	19.1 ± 2.6	38.3 ± 3.2 *	31.9 ± 6.9	36.6 ± 7.6 *	42.0 ± 3.6 *	42.1 ± 6.4 *

Note: *—significant difference from the ŽP strain (*t*-test, *p* ˂ 0.05).

**Table 5:** Characteristics of the tested APEC in IPEC strains in relation to the inhibition efficiency CFS of bacteriocin-producing *E. coli* strains.

Test-Strain ID	Pathotype	Insensitivity Profile		The Number of *E. coli* Strains Whose CFS Inhibited the Growth of Test-Strains after 22 h of Cultivation, %		
		To Antibiotics	To Bacteriocins	<20%	20–40%	>40%
BR4	APEC	Tet	A+B+D+E1-E7+Ia+Ib+K+N+M+S4+B17+C7+V	2	7	8
BR35	APEC	Amp+Cpf+Lfc+Gen+Tet	A+B+D+Ia+Ib+N+M+S4+B17+C7+V	0	5	12
BR37	APEC	Amp+Cpf+Cfo+Cft+Amk+Gen+Tet	A+B+D+E4+E5+Ia+Ib+K+N+M+C7+V	0	9	8
CA29	STEC/ETEC	-	not defined	8	9	0
CA43	STEC	Amp+Cfp+Cfp+Cfr+Azt	not defined	11	6	0
CA46	ETEC	Amp+Cfp+Cfr+Azt+Tet	not defined	10	7	0

Note: Amk—amikacin, Amp—ampicillin, Azt—aztreonam, Cfo—cefotaxime, Cfp—cefepim, Cfr—ceftriaxone, Cft—ceftazidim, Cpf—ciprofloxacin, Gen—gentamycin, Lfc—levofloxacin, Tet—tetracycline.

**Table 6:** Virulence-associated genes, presence of bacteriophages and hemolytic activity of bacteriocin-producing *E. coli* strains.

Strain	Virulence-Associated Genes													Bacteriophage	Hemolytic Activity
	*east1*	*ehxA*	*estI*	*estII*	*eltA*	*stx1*	*stx2*	*hlyA*	*hlyF*	*cnf1*	*fimH*	*afa/draBC*	*papC*		
Ch1	+	+	−	−	+	−	−	−	+	−	+	+	−	−	−
Q5	−	−	−	−	−	−	−	−	−	−	+	−	−	−	−
Q8	+	+	−	−	−	−	+	−	+	−	+	+	−	+	−
Q12	+	−	−	−	−	−	−	−	+	−	+	+	−	+	−
C18	−	−	−	−	−	−	−	−	−	−	+	−	−	+	−
C19	−	−	−	−	−	−	−	−	−	−	+	+	+	+	−
C23	−	−	−	−	−	−	−	−	−	−	+	−	+	−	−
C25	−	−	−	−	−	−	−	−	−	−	+	+	−	+	−
C32	−	−	−	−	−	−	−	−	−	−	+	+	−	+	−
C40	+	+	+	−	−	−	+	−	−	−	+	+	−	−	−
C41	−	−	−	−	−	−	−	−	−	−	+	−	−	−	−
C45	−	−	−	−	−	−	−	−	+	−	+	−	−	−	+
C48	−	−	−	−	−	−	−	−	+	−	+	+	−	−	−
C49	−	−	−	−	−	−	−	−	+	−	+	+	−	−	−
C51	−	−	−	−	−	−	−	−	+	−	+	+	−	+	−
C56	−	−	−	−	−	−	−	+	−	−	+	−	−	+	−
C61	−	−	−	−	−	−	−	+	−	−	+	+	+	−	−

Note: “+”—presence of a feature; “−”—absence of a feature.

## References

[1] Vila J., Sáez-López E., Johnson J.R., Römling U., Dobrindt U., Cantón R., Giske C.G., Naas T., Carattoli A., Martínez-Medina M. (2016). *Escherichia coli*: An old friend with new tidings. FEMS Microbiol. Rev..

[2] Oporto B., Ocejo M., Alkorta M., Marimón T., Montes M., Hurtado A. (2019). Zoonotic approach to Shiga toxin-producing *Escherichia coli*: Integrated analysis of virulence and antimicrobial resistance in ruminants and humans. Epidemiol. Infect..

[3] Maturana V.G., de Pace F., Carlos C., Pires M.M., de Campos T.A., Nakazato G., Stheling E.G., Logue C.M., Nolan L.K., da Silveira W.D. (2011). Subpathotypes of avian pathogenic *Escherichia coli* (APEC) exist as defined by their syndromes and virulence traits. Open Microbiol. J..

[4] Mageiros L., Méric G., Bayliss S.C., Pensar J., Pascoe B., Mourkas E., Calland J.K., Yahara K., Murray S., Wilkinson T.S. (2021). Genome evolution and the emergence of pathogenicity in avian *Escherichia coli*. Nat. Commun..

[5] Valdes A.M., Walter J., Segal E., Spector T.D. (2018). Role of the gut microbiota in nutrition and health: Discuss strategies for modulating the gut microbiota through diet and probiotics. BMJ.

[6] Pickard J.M., Núñez G. (2019). Pathogen colonization resistance in the gut and its manipulation for improved health. Am. J. Pathol..

[7] Mazurek-Popczyk J., Pisarska J., Bok E., Baldy-Chudzik K. (2020). Antibacterial activity of bacteriocinogenic commensal *Escherichia coli* against zoonotic strains resistant and sensitive to antibiotics. Antibiotics.

[8] Gordon D.M., O’Brien C.L. (2006). Bacteriocin diversity and the frequency of multiple bacteriocin production in *Escherichia coli*. Microbiology.

[9] Hammami R., Fernandez B., Lacroix C., Fliss I. (2013). Anti-infective properties of bacteriocins: An update. Cell. Mol. Life Sci..

[10] Rebuffat S., Drider D., Rebuffat S. (2011). Bacteriocins from Gram-Negative Bacteria: A Classification?. Prokaryotic Antimicrobial Peptides.

[11] Baquero F., Lanza V.F., Baquero M.-R., del Campo R., Bravo-Vázquez D.A. (2019). Microcins in *Enterobacteriaceae*: Peptide antimicrobials in the eco-active intestinal chemosphere. Front. Microbiol..

[12] Lagha A.B., Haas B., Gottschalk M., Grenier D. (2017). Antimicrobial potential of bacteriocins in poultry and swine production. Vet. Res..

[13] Askari N., Ghanbarpour R. (2019). Molecular investigation of the colicinogenic strains that are capable of inhibiting *E. coli* O157:H7 in vitro. BMC Vet. Res..

[14] Simons A., Alhanout K., Duval R.E. (2020). Bacteriocins, antimicrobial peptides from bacterial origin: Overview of their biology and their impact against multidrug-resistant bacteria. Microorganisms.

[15] Cameron A., Zaheer R., Adator E.H., Barbieri R., Reuter T., McAllister T.A. (2019). Bacteriocin occurrence and activity in *Escherichia coli* isolated from bovines and wastewater. Toxins.

[16] Zaslavskaya M.I., Makhrova T.V., Aleksandrova N.A., Ignatova N.I., Belova I.V., Tochilina A.G., Solovyeva I.V. (2019). Prospects for using bacteriocins of normal microbiota in antibacterial therapy (review). Sovrem. Tehnol. V Med..

[17] Versalovic J., Koeuth T., Lupski J.R. (1991). Distribution of repetitive DNA sequences in eubacteria and application to fingerprinting of bacterial genomes. Nucleic Acids Res..

[18] Kuznetsova M.V., Gizatullina J.S., Nesterova L.Y., Starčič Erjavec M. (2020). *Escherichia coli* isolated from cases of colibacillosis in Russian poultry farms (Perm Krai): Sensitivity to antibiotics and bacteriocins. Microorganisms.

[19] Mihailovskaya V.S., Artamonova O.A., Zhdanova I.N., Kuznetsova M.V. Prevalence of pathogenicity determinants among *Escherichia coli* strains isolated from healthy cows and calves in farms of Perm Krai. Mechanisms of microorganisms adaptation to different habitat condition. Proceedings of the All-Russian Scientific Conference with International Participation.

[20] Budič M., Rijavec M., Petkovšek Z., Žgur-Bertok D. (2011). *Escherichia coli* bacteriocins: Antimicrobial efficacy and prevalence among isolates from patients with bacteraemia. PLoS ONE..

[21] Starčič Erjavec M., Petkovšek Ž., Kuznetsova M., Maslennikova I., Žgur-Bertok D. (2015). Strain ŽP—The first bacterial conjugation-based «kill»—«anti-kill» antimicrobial system. Plasmid.

[22] Šmajs D., Micenková L., Šmarda J., Vrba M., Sevčíková A., Vališová Z., Woznicová V. (2010). Bacteriocin synthesis in uropathogenic and commensal *Escherichia coli*: Colicin E1 is a potential virulence factor. BMC Microbiol..

[23] Chapman T.A., Wu X.-Y., Barchia I., Bettelheim K.A., Driesen S., Trott D., Wilson M., Chin J.J.-C. (2006). Comparison of virulence gene profiles of *Escherichia coli* strains isolated from healthy and diarrheic swine. Appl. Environ. Microbiol..

[24] Moulin-Schouleur M., Répérant M., Laurent S., Brée A., Mignon-Grasteau S., Germon P., Rasschaert D., Schouler C. (2007). Extraintestinal pathogenic *Escherichia coli* strains of avian and human origin: Link between phylogenetic relationships and common virulence patterns. J. Clin. Microbiol..

[25] Micenková L., Bosák J., Štaudová B., Kohoutová D., Čejková D., Woznicová V., Vrba M., Ševčíková A., Bureš J., Šmajs D. (2016). Microcin determinants are associated with B2 phylogroup of human fecal *Escherichia coli* isolates. Microbiologyopen.

[26] Riley M.A., Gordon D.M. (1992). A survey of Col plasmids in natural isolates of *Escherchia coli* and an investigation into the stability of Col plasmid lineages. J. Gen. Microbiol..

[27] Feldgarden M., Riley M.A. (1999). The phenotypic and fitness effects of colicin resistance in *Escherichia coli* K12. Evolution.

[28] Braun V., Patzer S.I., Hantke K. (2002). Ton-dependent colicins and microcins: Modular design and evolution. Biochimie.

[29] Murinda S.E., Roberts R.F., Wilson R.A. (1996). Evaluation of colicins for inhibitory activity against diarrheagenic *Escherichia coli* strains, including serotype O157:H7. Appl. Environ. Microbiol..

[30] FAO/WHO (2002). Joint F.A.O./W.H.O. (Food and Agriculture Organization/World Health Organization) Working Group Report on Drafting Guidelines for the Evaluation of Probiotics in Food.

